# Health and Household Air Pollution from Solid Fuel Use: The Need for Improved Exposure Assessment

**DOI:** 10.1289/ehp.1206429

**Published:** 2013-07-19

**Authors:** Maggie L. Clark, Jennifer L. Peel, Kalpana Balakrishnan, Patrick N. Breysse, Steven N. Chillrud, Luke P. Naeher, Charles E. Rodes, Alan F. Vette, John M. Balbus

**Affiliations:** 1 Department of Environmental and Radiological Health Sciences, Colorado State University, Fort Collins, Colorado, USA; 2 Department of Environmental Health Engineering, Sri Ramachandra University, Porur, Chennai, India; 3 Environmental Health Sciences, Johns Hopkins University, Baltimore, Maryland, USA; 4 Lamont-Doherty Earth Observatory of Columbia University, Palisades, New York, USA; 5 Department of Environmental Health Science, University of Georgia, Athens, Georgia, USA; 6 Research Triangle Institute, Research Triangle Park, North Carolina, USA; 7 U.S. Environmental Protection Agency, Research Triangle Park, North Carolina, USA; 8 National Institute of Environmental Health Sciences, National Institutes of Health, Department of Health and Human Services, Bethesda, Maryland, USA

## Abstract

Background: Nearly 3 billion people worldwide rely on solid fuel combustion to meet basic household energy needs. The resulting exposure to air pollution causes an estimated 4.5% of the global burden of disease. Large variability and a lack of resources for research and development have resulted in highly uncertain exposure estimates.

Objective: We sought to identify research priorities for exposure assessment that will more accurately and precisely define exposure–response relationships of household air pollution necessary to inform future cleaner-burning cookstove dissemination programs.

Data Sources: As part of an international workshop in May 2011, an expert group characterized the state of the science and developed recommendations for exposure assessment of household air pollution.

Synthesis: The following priority research areas were identified to explain variability and reduce uncertainty of household air pollution exposure measurements: improved characterization of spatial and temporal variability for studies examining both short- and long-term health effects; development and validation of measurement technology and approaches to conduct complex exposure assessments in resource-limited settings with a large range of pollutant concentrations; and development and validation of biomarkers for estimating dose. Addressing these priority research areas, which will inherently require an increased allocation of resources for cookstove research, will lead to better characterization of exposure–response relationships.

Conclusions: Although the type and extent of exposure assessment will necessarily depend on the goal and design of the cookstove study, without improved understanding of exposure–response relationships, the level of air pollution reduction necessary to meet the health targets of cookstove interventions will remain uncertain.

Citation: Clark ML, Peel JL, Balakrishnan K, Breysse PN, Chillrud SN, Naeher LP, Rodes CE, Vette AF, Balbus JM. 2013. Health and household air pollution from solid fuel use: the need for improved exposure assessment. Environ Health Perspect 121:1120–1128; http://dx.doi.org/10.1289/ehp.1206429

## Introduction

Nearly 3 billion people worldwide, and a great majority of households in developing countries, rely on solid fuels (such as wood, dung, crop residues, coal, and charcoal) with little or no access to modern fuels for cooking and other household energy needs (Lim et al. 2012; Smith et al. 2012). In these households, solid fuels are often burned in inefficient, poorly vented combustion devices (open fires, traditional stoves). The incomplete combustion of these solid fuels results in much of the fuel energy being emitted as potentially toxic pollutants, including particles of varying sizes, carbon monoxide (CO), nitrogen dioxide, volatile and semivolatile organic compounds (e.g., formaldehyde and benzo[*a*]pyrene), methylene chloride, and dioxins (Naeher et al. 2007). Combustion of coal, in addition to the above pollutants, releases sulfur oxides, heavy metals such as arsenic, and fluorine [World Health Organization (WHO) 2006]. The use of solid fuels, primarily for cooking, has been estimated to be responsible for > 3.5 million premature deaths per year (plus an additional 0.5 million deaths from outdoor air pollution due to household fuel use) and 110 million disability-adjusted life years (DALYs) (Lim et al. 2012).

Several large-scale initiatives are under way for the dissemination of cleaner-burning stoves (Martin et al. 2011); however, the stoves being disseminated may not achieve the desired exposure reductions and health benefits given the lack of robust exposure–response information. These high-profile efforts are building needed momentum and bringing financial support and attention to this important global health issue. For example, the Global Alliance for Clean Cookstoves (GACC) is a public-private partnership led by the United Nations Foundation whose goal is for 100 million homes worldwide to adopt clean, efficient stoves and fuels by 2020 (GACC 2012). In the face of immense practical and cultural barriers to sustainable and effective cookstove interventions, for this effort and others like it to be successful in the design and dissemination of cleaner-burning cookstoves that will meaningfully improve health, the fundamental question “How clean is clean enough?” must be answered. Despite this knowledge gap, an international workshop consisting of 91 stakeholders (cookstove manufacturers, disseminators, researchers, and academics) from 23 countries developed a guidance policy on emissions testing and voluntary standards for improved cookstoves (International Standards Organization 2012). Although the policy relies on a set of tiers for exposure reduction rather than specifying a health-based emissions standard, it does note the need to incorporate the results of future studies to specify such a health-based standard.

The extreme variability within and between personal exposures to cookstove-related air pollution, as well as multiple sources of exposure measurement error, are major sources of uncertainty around the exposure–response curve. For example, Smith et al. (2011) reported the first exposure–response evaluation within a cookstove intervention study; the results illustrated the difficulty in estimating health outcome improvements from specific intervention-related exposure reductions. A 50% reduction in personal CO exposure comparing group means for the control and intervention arms of the trial was associated with an estimated 18% reduction in risk for physician-diagnosed pneumonia in children; however, the 95% confidence interval (CI) suggests that these data are consistent with a risk reduction that ranges from 2% to 30% (Smith et al. 2011).

In May 2011, an international workshop, “Health Burden of Indoor Air Pollution on Women and Children in Developing Countries,” led by the National Institutes of Health, convened > 150 participants to review the state of the science regarding the health impacts of exposures to air pollution from the household use of solid fuels including indoor, near household, and outdoor environments [household air pollution (HAP)]. Acknowledging the considerable progress achieved to date by previous research, the workshop’s Exposure Assessment and Biomarkers Working Group identified several research priorities as potentially having the biggest impact on the cookstove field, focusing specifically on information needed to better inform stove dissemination programs. The working group discussed questions regarding critical site-specific design choices, such as the duration of the measurement (e.g., cooking period, 24 hr, 48 hr), the number of repeated measures necessary to characterize temporal variability, and the monitor type (area vs. personal) and placement necessary to characterize spatial variability; the relevant pollutants of interest; as well as the appropriate methods to estimate pollutant dose. Here, we summarize the existing state of the knowledge, identify gaps, and provide recommendations for exposure assessment research needed to answer the question “How clean is clean enough?”

## Issues: Exposure Assessment Approaches and Research Priorities

**Figure 1:**
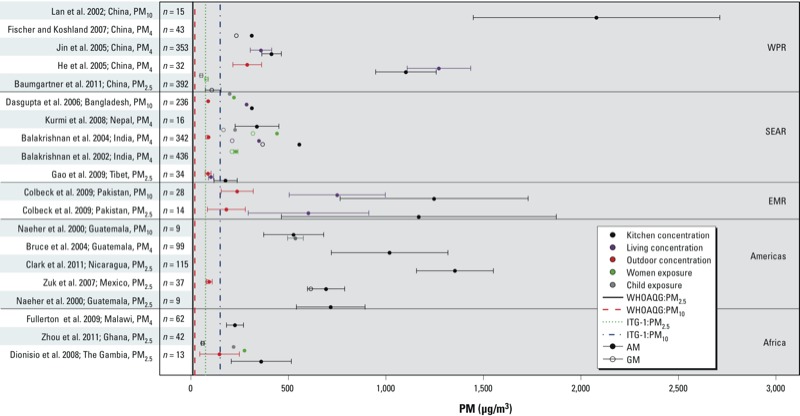
Reported means ± SDs of 24-hr PM (PM_10_, PM_4_, and PM_2.5_) concentrations and/or exposures (μg/m^3^) from selected studies included in the WHO Global household air pollution measurement database (http://www.who.int/indoorair/health_impacts/databases_iap/en/). Pollutant-specific WHO interim and guideline values, respectively, for air quality displayed refer to the annual guidelines of 70 μg/m^3^ and 10 μg/m^3^ for PM_10_ and 35 μg/m^3^ and 10 μg/m^3^ for PM_2.5_ (WHO 2006). Studies are labeled according to the reference, country, and reported PM fraction. For some studies reporting mean levels across multiple categories, such as season or fuel/kitchen type, results are shown as the pooled means and pooled SDs. Abbreviations: AM, arithmetic mean; EMR, Eastern Mediterranean Region; GM, geometric mean; ITG-1, interim target guideline; PM_4_, ≤ 4 μm in aerodynamic diameter; PM_10_, ≤ 10 μm in aerodynamic diameter; SEAR, Southeast Asian Region; WHOAQG, World Health Organization Air Quality Guideline; WPR, Western Pacific Region.

**Figure 2:**
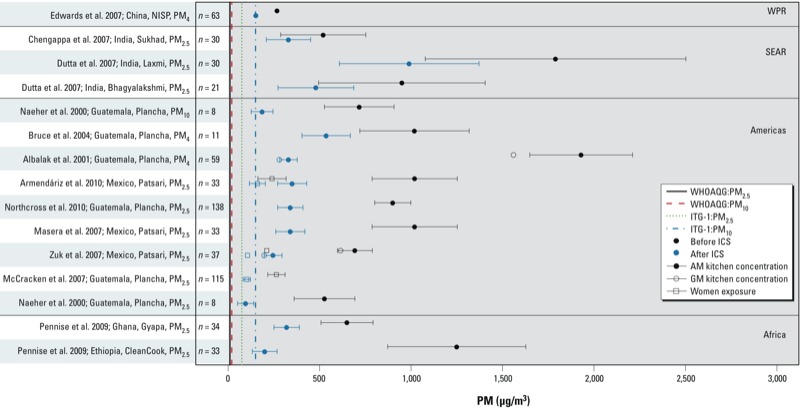
Reported means ± SDs from selected studies included in the WHO Global household air pollution measurement database that measured PM concentrations and/or exposures (μg/m^3^) before and after the introduction of an improved-combustion cookstove (http://www.who.int/indoorair/health_impacts/databases_iap/en/). Studies are labeled according to the reference, country, cookstove used, and reported PM fraction. Pollutant-specific WHO interim and guideline values, respectively, for air quality refer to the annual guidelines of 70 μg/m^3^ and 10 μg/m^3^ for PM_10_ and 35 μg/m^3^ and 10 μg/m^3^ for PM_2.5_ (WHO 2006). Abbreviations: AM, arithmetic mean; GM, geometric mean; ICS, improved combustion stove; ITG-1, interim target guideline; NISP, National Improved Stove Program; PM_4_, ≤ 4 μm in aerodynamic diameter; PM_10_, ≤ 10 μm in aerodynamic diameter; SEAR, Southeast Asian Region; WHOAQG, World Health Organization Air Quality Guideline; WPR, Western Pacific Region.

Increasing evidence from household air pollution studies conducted in developing countries points to the complexity and heterogeneity in exposure patterns [a comprehensive summary of study results is available in the WHO Global Household Air Pollution Measurement Databases (http://www.who.int/indoorair/health_impacts/databases_iap/en/)]. Figure 1 displays 24-hr area and outdoor particulate matter (PM) concentrations as well as personal exposures reported by selected studies from the WHO database; these studies were chosen to highlight various regions of the world and to demonstrate several of the following pertinent exposure assessment issues. Household air pollution concentrations (including outdoor concentrations in communities affected by inefficient biomass and coal combustion emissions) are consistently much higher than the annual WHO air quality guideline values and, in most instances, exceed the higher 24-hr interim target guideline concentrations. “Improved,” cleaner-burning stove designs have the potential to substantially reduce air pollution emissions and exposures (Smith 2002). Figure 2 presents results from several studies that measured PM concentrations in kitchens or personal exposures before and after the introduction of improved-combustion cookstoves. In most instances, substantial reductions were observed compared with baseline; however, the concentrations reported postintervention continue to remain well above WHO guideline values. In Figures 1 and 2, the pollutant concentrations are not only extremely high but are also characterized by large variability that can be attributed to a myriad of factors (e.g., variability in cookstove use and time–activity patterns, weather conditions, household room configuration and ventilation, fuel type and moisture, instrument error). The nature and type of measurements performed, including the choice of PM size fraction, have depended on the specific objectives of the individual studies and are often limited by financial and logistical constraints imposed by settings in developing countries. Thus, although Figures 1 and 2 are not meant to provide a comprehensive review of cookstove studies, the figures demonstrate the considerable uncertainty that exists regarding the nature and magnitude of exposure variability across all pertinent regions of the world, a factor that has made direct comparisons across studies challenging and that makes the choice of a new cleaner-burning stove technology difficult.

Although exposure assessment methods in cookstove research have progressed over the past decade, there have been limited gains in reducing the uncertainties in the exposure–response relationship and determining the reductions in concentrations needed to improve health. The type of exposure assessment conducted needs to be determined within the scope and goals of a particular study (i.e., chronic disease epidemiologic studies may not require highly time-resolved exposure assessment); however, this discussion focuses on the accuracy and precision of various exposure assessment approaches and the resulting contributions these methods may have in order to better characterize exposure response and inform large-scale stove dissemination programs. Within this context, Table 1 summarizes the advantages and disadvantages of selected exposure assessment approaches used in studies examining the impact of cookstove emissions on health as well as considerations for implementation that are further described below. Many health studies to date have used qualitative measures of household air pollution exposure, such as fuel or stove type (Table 1, method A); this approach is inexpensive but has several serious limitations primarily relating to exposure misclassification. Although some information on exposure variation within fuel/stove type can be gained with the addition of semiquantitative measures (e.g., incorporating time activity; Table 1, method B), these approaches have limited utility for quantifying robust exposure–response relationships.

**Table 1:** Summary of selected exposure assessment methods used in studies examining the impact of cookstove emissions on health and specific implications regarding the contribution each method may have on the ability to answer the question “How clean is clean enough?” for large-scale stove dissemination programs.

Exposure assessment method	Advantages	Disadvantages	Implications and comments
A. Fuel/stove type	Low cost per household allowing for larger sample size than other methods	Large variation in exposure within fuel and stove types; no exposure–response information; mixed use of fuel/stove types can lead to exposure misclassification	Questionable contribution to scientific knowledge/policy for maximizing the public health benefits of stove dissemination programs
B. Fuel/stove type with additional semi­quantitative measures (e.g., time activity, cooking behavior, fuel quality, stove condition, and ventilation)	Relatively low cost per household allowing for larger sample size than other methods; explain more of the exposure variation within fuel/stove type than method A	No exposure–response information; subjective nature of additional semi­quantitative measures can lead to exposure misclassification	Same as method A; utility (e.g., variation explained) depends heavily on site-specific characteristics
C. Area/kitchen pollutant concentrations	Exposure response can be characterized if area concentration represents true long-term personal exposure; area measures may serve as accurate indicators of long-term exposure when substantial daily variation exists within personal measures; less invasive compared with personal exposure; less dependent on subject compliance than personal exposure	Personal exposure not captured due to variation in behavioral characteristics (e.g., time–activity patterns); lack of affordable, time-resolved instruments that are accurate for a wide range of pollutant concentrations; more expensive than fuel/stove type assignment	Adequate temporal resolution is unknown (e.g., length of measure, number of repeated measures, and seasonality) and will depend on study design and objective; need to consider horizontal and vertical concentration gradients when placing instruments; PM and CO are typically measured, lacking information about entire pollutant mix
D. Area/kitchen pollutant concentrations with additional semi­quantitative measures (e.g., time activity, cooking behavior, other pollutant sources)	Same as method C; allows for estimation of personal exposure	Lack of affordable, time-resolved instruments that are accurate for a wide range of pollutant concentrations; more expensive than fuel/stove type assignment; subjective nature of additional semi­quantitative measures can lead to personal exposure misclassification	Same as method C; utility (e.g., variation explained) depends heavily on site-specific characteristics
E. Personal pollutant concentrations	Integrates exposure over space and time; considered the gold standard when participant compliance is optimized and the appropriate temporal scale is identified and captured; exposure response can be characterized if personal concentration is the exposure of interest; objective method to capture variation in behavioral characteristics; with real-time instruments, data on both exposure concentrations and patterns of exposure can be measured	Lack of lightweight, affordable, time-resolved instruments that are accurate for a wide range of pollutant concentrations; more expensive than fuel/stove type assignment; difficult to employ among children or sick adults; more dependent on subject compliance than area measurements	Adequate temporal resolution is unknown (e.g., length of measure, number of repeated measures, and seasonality) and will depend on study design and objective; PM and CO are typically measured, lacking information about entire pollutant mix; will capture exposure from non-cookstove sources (e.g., ambient air pollution, secondhand smoke)
F. Combined area/kitchen and personal pollutant concentrations^*a*^	Exposure response can be characterized; relationship between area/kitchen and personal exposure concentrations can be characterized	Expensive and time intensive	Adequate temporal resolution is unknown (e.g., length of measure, number of repeated measures, seasonality) and will depend on study design and objective; relationship between area/kitchen and personal exposure concentrations are likely site and season specific
G. Internal dose/biomarker of exposure	Reflects absorbed dose accounting for interindividual differences in factors (e.g., breathing rate and ventilation volume, host factors affecting susceptibility)	Dependent on temporal nature of biomarker (e.g., half-life); may not be source specific	Reliable and accurate biomarkers for household air pollution exposures have not been identified and validated; choice of biomarker will depend on the study question (e.g., acute versus chronic effects); choice of biomarker could influence cost and level of invasiveness
^***a***^Addresses advantages, disadvantages, and comments for the combination of area and personal methods; it does not refer to these issues associated with using only area methods or only personal methods.			

Table 1 further compares benefits, limitations, and knowledge gained by conducting various types of quantitative exposure assessments (methods C–G). Cooking area pollution concentrations can provide a quantified measure of the environment (Table 1, method C); however, these area concentrations fail to capture personal exposure measures because of large spatial heterogeneity and differences in time–activity patterns. Using area concentrations to estimate personal exposure will likely result in considerable uncertainty in exposure–response assessments. An advantage of the area-monitoring approach is that measurements from a subset of homes can be used in conjunction with air exchange rates and building characteristics to estimate area concentrations for households without area monitoring data. However, these predicted area concentrations are affected by potential uncertainty in the estimated air exchange rates. As with method B (Table 1), the addition of time–activity information can better capture the variation due to individual behavior (Table 1, method D). In contrast, personal exposure measurements (Table 1, method E) incorporate individual behavior without relying on subjective methods. However, there is a trade-off between capturing short-term and long-term exposure variability. Although an expensive and time-intensive option, combining area/kitchen and personal pollutant measures allows the relationship between personal and area/kitchen measurements to be characterized (Table 1, method F). For example, comprehensive area and personal exposure measurements among a subgroup of the target population can be used to develop exposure models to estimate personal exposures in the absence of personal monitoring data in the larger study population. However, an important limitation is that these types of exposure models should be applied only to the same target population for which the detailed exposure monitoring was conducted. Finally, the development of validated and source-specific biomarkers may provide the opportunity to assess dose at the individual level (Table 1, method G).

*Characterizing spatial and temporal variability*. Many factors affect the ability to accurately characterize variability of true personal exposure. To be comprehensive, exposure assessments should characterize the magnitude, frequency, and duration of exposure. As discussed above, personal exposure can be estimated using area measurements of pollutant concentrations and time–activity information, or by directly placing the sampler on the person. Area samples are subject to variability due to temporal and spatial gradients around a cookstove. Generalizability of these measures requires detailed characterization of this variability and the time–location of the subjects. A personal air pollution sample, on the other hand, is a complex assessment that by definition integrates exposure over space and time; when participant compliance is optimized and the appropriate temporal scale is identified and captured, personal exposure sampling is considered the gold standard for true personal exposure (Rodes and Thornburg 2012). Generalizability of personal samples requires a detailed characterization of the determinants of exposure that allow a model to explain both within-person and between-person variability.

Real-time and time-integrated approaches can be used to assess personal exposures. Time-integrated sampling provides an average value over the time sampled. Real-time measures of pollutant concentration can constitute a rich data set for assessing temporal variability. The length of the sampling time is a crucial consideration in assessing temporal variability for both approaches and will change depending on the purpose of sampling (e.g., whether to describe long-term or short-term exposure) and the site characteristics and behavior of the study population. For example, a personal exposure measurement during a single cooking event will provide an assessment while cooking; a 24-hr measure will provide an assessment over multiple cooking events and will integrate non-cooking time exposures to emissions from other sources. Multiple-day measures allow assessment of variability of both cooking and non-cooking time exposures across an even larger sampling of cooking events and days.

Behavioral patterns and individual-level characteristics (e.g., age, socioeconomic status, time spent in the cooking area) and household-level characteristics (e.g., fuel/stove type, cookhouse ventilation, use of biomass for heating, location of the kitchen in relation to other rooms) may all contribute to variations in personal exposures, both within and between individuals. In addition, these characteristics and thus the magnitude of exposures will vary across geographical regions (countries, cities, villages, and neighborhoods), time, and season of the year. These characteristics contribute to the within-subject variability in personal exposures. Studies have demonstrated that within-subject CO exposure variability over time was about three times greater than between-subject CO exposure variability (Dionisio et al. 2012; McCracken et al. 2009). The magnitude of within-subject variability underscores the importance of determining the necessary number of repeated exposure measurements to accurately characterize exposures over time. Because attenuation bias in exposure–response relationships increases with the ratio of the within variance component to the between variance component (Brunekreef et al. 1987; Rappaport and Kupper 2004), it is important to accurately estimate these variance components in studies of health effects. If resources and logistics prohibit obtaining numerous repeated measures for the entire study population, measurements on a subset would allow the magnitude of attenuation bias to be estimated and also permit adjustment for this bias in the exposure–response characterization (Armstrong 1998).

Depending on the health end point of interest (e.g., acute or chronic disease), studies need to assess exposures over different time scales. Studies evaluating acute health end points, such as acute episodes of acute lower respiratory infection (ALRI) in children (Bautista et al. 2009; Smith et al. 2011), need to carefully assess the frequency of exposure over a short duration (e.g., same day, 2 weeks before the episode). In the acute studies, the appropriate exposure window needs to be defined and personal exposure within this window needs to be characterized. Particularly for acute health responses, integrated (i.e., 24-hr mean) exposure assessments may not be sufficient and will likely underestimate true exposure (Ezzati and Kammen 2001). Accurately characterizing episodes of high-intensity exposure (i.e., peaks) may be necessary for defining exposure response.

The goal of studies focusing on chronic diseases is to estimate long-term exposures; measurements are intended to reflect typical exposures over many years. For example, determining chronic obstructive pulmonary disease (COPD) or cancer risk will require estimates of lifetime exposure. Few studies have attempted to address what exposure assessment methods are needed to reflect typical long-term exposures. In Guatemala, McCracken et al. (2009) reported that combining a single 48-hr personal CO exposure measure with descriptive information (e.g., stove type, residential characteristics) better predicted long-term CO exposure among children than a single 48-hr personal CO exposure measure alone. These results indicate that, if limited to a single personal measure, descriptive information on important exposure determinants can improve long-term exposure estimation. Also in the context of estimating long-term exposures, McCracken et al. (2013) evaluated repeated measures of personal CO as a surrogate of repeated measures of personal PM_2.5_ (≤ 2.5 μm in aerodynamic diameter). In a setting where biomass cookstove combustion is the dominant source of pollution and pollution concentrations are relatively high for both traditional stove users and improved stove users, personal CO explained 78% of the between-person variation in personal PM_2.5_ (McCracken et al. 2013). McCracken et al. (2009, 2013) have successfully illustrated some of the nuances surrounding exposure assessment design decisions. However, further research is needed to determine whether the long-term relationship between personal CO and PM_2.5_ is valid, especially at lower pollutant concentrations and in areas with multiple pollutant sources, and also to identify what the minimal sufficient measuring period or number of repeated measures should be in order to capture typical long-term exposures; this decision will likely be site and population specific.

Cooking area concentrations could provide additional resolution for estimating long-term personal exposures. However, the value of area measures largely depends on differences in individual behavior over time (e.g., time–activity patterns) and can also be influenced by horizontal and vertical concentration gradients in the cooking areas (Kar et al. 2012). Studies examining the correlation of personal and area measures have been inconsistent (Naeher et al. 2007). Cynthia et al. (2008) suggest that using the reduction in area measures as a surrogate for reductions in personal exposures may not be valid for evaluating interventions. In addition, Armendáriz-Arnez et al. (2010) reported different relationships between personal PM_2.5_ and indoor PM_2.5_ for open fire as compared with Patsari improved stove users in Mexico. Further research is necessary to determine whether area exposure concentrations can be used to estimate personal exposures or even serve as better indicators of typical long-term exposure as compared to a limited number of personal exposure samples. If fewer repeated area measures are needed to explain more of the variation in usual long-term exposures as compared with personal measures, this could have far-reaching impacts on costs, participation, and other logistical concerns of long-term intervention studies.

In communities that rely heavily on solid fuels, household emission of pollutants can be an important contributor to ambient air pollution (Chafe et al. 2011). As a result, these communities often suffer from both elevated indoor and outdoor air pollution. Further, household-level gains in reducing kitchen-area concentrations of pollutants through ventilation may be offset by increasing ambient pollution concentrations. Tangible reductions in personal exposures could thus require fuel/stove interventions for the entire community rather than for a limited number of households within the community (Chowdhury et al. 2012). Accordingly, the role of exchange between the outdoor and indoor microenvironments in influencing area concentrations and personal exposures needs to be better explored. Alternatively, communities in warmer, drier climates (e.g., Africa) often cook on stoves located outside of the house/kitchen structure. Although cooking outdoors will result in more rapid pollutant dispersion compared with cooking indoors, the intimate interaction between the cook, and potentially children, and the stove could still result in excessive exposures during meal preparation.

Adoption and sustained use of a cookstove intervention can also contribute to the temporal variability in exposure. Although many studies are beginning to report results from programmatic intervention efforts to distribute improved cookstoves (Masera et al. 2007; Smith et al. 2007), it is crucial to understand how short-term exposure measurements may relate to the uptake and sustained use of a new technology. Transition to a new stove technology has been shown to be a dynamic process, with usage patterns changing over time and involving multiple stoves (Pine et al. 2011; Ruiz-Mercado et al. 2011). Therefore, adoption and use also need to be considered when characterizing variation in personal exposure. In addition, the timing of the exposure measurements relative to the stove introduction should be taken into account to allow time for the transition to the new stove technology (Pine et al. 2011; Ruiz-Mercado et al. 2011).

*Evaluating the complex pollutant mixture.* The amount and relative proportion of air pollutants generated by solid fuel combustion are dependent on a number of factors, including fuel type and moisture content, household ventilation, the behavior of the people using the stoves, and the stove technology (Fullerton et al. 2008; Smith 2002). For example, more efficient stoves with higher combustion temperatures may decrease PM_2.5_ emissions overall compared with open fires but actually increase other potentially more toxic emissions such as ultrafine PM, black carbon, and polycyclic aromatic hydrocarbons (PAHs) (e.g., Koziński and Saade 1998; L’Orange et al. 2012; Lu et al. 2009). Similarly, charcoal burning may reduce PM but increase CO exposures (Ellegard 1996). Furthermore, laboratory-based measurements of composition changes in stove emissions as a function of fuel type and moisture content, stove type, combustion temperature and efficiency, and other factors may or may not translate into real-world settings. In addition, the type and range of pollutants of interest may vary depending on the health end point of interest. Measuring a single pollutant may thus not serve as an adequate surrogate for other pollutants. In contrast to the results described above from McCracken et al. (2013), Dionisio et al. (2012) reported a poor correlation between an indirect method to estimate personal PM_2.5_ exposure (applying the relationship between cooking-area PM_2.5_ and CO to personal CO measures) and directly measured personal PM_2.5_ among children in The Gambia. These results indicate that, in a setting that may have multiple sources of pollution, 48-hr personal CO measures may not be an adequate proxy for personal PM_2.5_ exposure.

Given the consistency of effects observed across PM sources and doses [e.g., as demonstrated for cardiovascular mortality by Pope et al. (2009) and Smith and Peel (2010)], PM_2.5_ mass may be the most relevant parameter of interest for health; however, this is not currently known given the lack of measurements of pollutants other than PM_2.5_ and CO. Much of the emphasis for health effects has been on PM_2.5_ because it is thought to be the most relevant size fraction for health. However, limited evidence exists in the ambient air pollution literature regarding the health effects of coarse and ultrafine PM (Brunekreef and Forsberg 2005; Health Effects Institute 2013). Furthermore, lung deposition varies by factors such as age, sex, breathing rate, underlying disease state, and PM aerodynamic size fraction (Chalupa et al. 2004; Goldberg and Lourenco 1973; Kim and Hu 2006; Kim and Kang 1997; Ruzer and Harley 2013; Segal et al. 2002). Little is known about how cooking fuel and combustion characteristics change particle size distribution and composition in real-world settings. Therefore, further work is needed to elucidate the relevant pollutants from biomass and coal combustion and their relative toxicity.

Interactions with behavioral factors may also complicate exposures to multiple pollutants. For example, reductions in irritant gases in cookstove emissions may paradoxically lead to increased exposures to other toxic constituents if, as a result, cooks and children spend more time closer to the fire. Therefore, the extent to which PM or CO may serve as proxies for each other or for other toxics remains to be elucidated and will also likely depend on the number and magnitude of other nearby pollutant sources. Source apportionment is a tool that could be used to distinguish pollutant sources, thereby reducing exposure misclassification.

In addition, many of the devices currently used to measure air pollution were designed for use in developed countries where concentrations are typically two to three orders of magnitude lower than those encountered in indoor biomass combustion environments. Additional biases and uncertainties are introduced in accurately describing the dose–response relationship without having an instrument that can be used across a wide range of air pollutant concentrations. In addition, many existing instruments, particularly those for measuring pollutants other than PM and CO, cannot feasibly be used in settings typical of the rural areas of developing countries because of limited portability and durability of instrumentation. Similarly, many currently available instruments were designed for use during an 8-hr work day in an industrial setting, which introduces additional obstacles for cookstove exposure assessment when the need is to deploy instruments for at least 24 hr and, potentially, for several consecutive days. Finally, the choice of exposure measurement methods and instrumentation needs to consider challenges associated with working in developing counties, including nonexistent or intermittent electrical power, remote locations, the lack of traditional laboratory space for equipment maintenance and calibration, and security issues.

*Developing methods to estimate dose.* Air pollution measurements may not adequately reflect the absorbed dose for the individual because of interindividual differences in routes of exposure and physiological factors such as breathing rate and ventilation volume, metabolism, and excretion. This limitation may be addressed by the development and use of appropriate biomarkers. However, few biomarkers have been assessed or validated to address household air pollution exposures. Further work is needed to identify the appropriate timing of effect, particularly for biomarkers with short half-lives (Simpson and Naeher 2010). Studies have reported the use of exhaled CO or carboxyhemoglobin, hydroxylated PAHs (OH-PAHs), methoxyphenols, and levoglucosan as potential biomarkers for biomass smoke exposure. OH-PAHs are the most commonly used, and seem to show good responses in the exposure settings relevant to the residential combustion of biomass fuel in developing countries. However, other pollutant sources could influence the results because biomass smoke is not the unique source of the parent compounds of these biomarkers. The other classes of biomarkers, methoxyphenols and levoglucosan, are also not unique to biomass smoke (e.g., there are also dietary sources). Validation field studies are necessary to determine the correlations between biomarkers of exposure and measured personal exposure concentrations in both the acute and chronic settings.

In addition to identifying and further characterizing biomarkers of exposure, more accurately estimating breathing rates and ventilation volume may reduce measurement error when combined with personal exposure measures. Recent advances in integrating accelerometry into miniaturized real-time air pollutant monitors (Rodes et al. 2012) could make these estimates more feasible in field settings typical of developing countries. More research is needed to determine whether these various methods of estimating dose could be complementary, or if breathing rate and ventilation volume in combination with personal exposures may actually be more accurate than short-lived biomarkers of exposure in certain circumstances, such as for estimating long-term exposure.

*Generating exposure–response functions.* As previously discussed, inadequate assessment of exposure–response relationships and exposure determinants makes it difficult to define the degree of exposure reduction necessary to achieve health benefits (Ezzati and Kammen 2001; Ezzati et al. 2000). In addition, the shape of the curve is critical, particularly for nonlinear exposure–response relationships (Martin et al. 2011). Smith and Peel (2010) observed that the exposure–response relationship for PM and cardiovascular disease mortality risk (Pope et al. 2009), which begins to plateau at relatively high PM exposures, implies that exposures need to be reduced to levels typically experienced in relatively clean ambient environments in order to achieve large-scale public health benefits for cookstove interventions. Pope et al. (2011) have developed similar exposure–response curves for lung cancer and cardiopulmonary mortality; the shape for cardiopulmonary mortality was similar to that for cardiovascular disease described above, but the shape for lung cancer was nearly linear. Given that different diseases (mortality as well as morbidity) may exhibit varying exposure–response patterns; the answer to the question “How clean is clean enough?” will depend on the evidence across the spectrum of disease end points.

Two recent large-scale randomized trials evaluating the impact of a cleaner burning stove intervention on exposure and health among women and children have illustrated the importance of strong quantitative exposure assessment when assessing health impacts (Romieu et al. 2009; Smith et al. 2011). Stronger associations with health end points were demonstrated when using measured pollutant concentrations or time-varying categories of stove use as compared with intention-to-treat analyses (analogous to using stove type as the exposure proxy), likely due to the exposure misclassification introduced when assuming complete and sustained adoption of the intervention cookstove.

**Figure 3:**
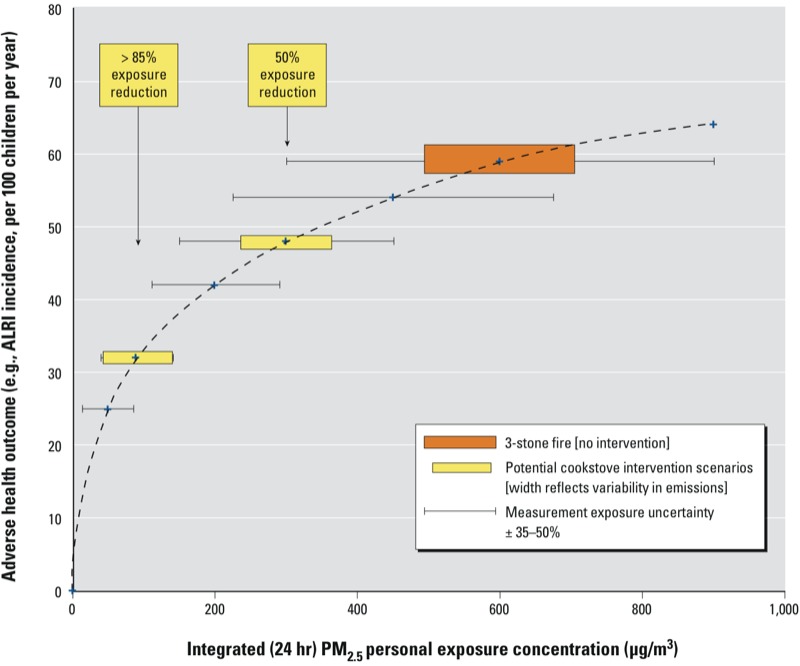
Hypothetical exposure–response relationship between PM_2.5_ and ALRI that illustrates how uncertainty in exposure assessment can limit the ability to accurately define the exposure reduction resulting from an intervention and, therefore, the true shape of the exposure–response curve.

Figure 3 presents a theoretical exposure–response curve that highlights how measurement uncertainty of exposure instruments limits our ability to accurately and precisely define exposure reductions resulting from cookstove interventions, as well as determine the shape of the exposure–response curve. It illustrates the need for a range of exposure concentrations to reasonably define the true shape of the curve (examples presented are for varying degrees of health improvements associated with cookstove interventions that reduce exposure concentrations by both 50% and 85%). Figure 3 also demonstrates the need to reduce instrument measurement uncertainty in order to identify how effective specific stove interventions are in reducing exposures and, thus, improving health. For example, in the hypothetical curve in Figure 3, if a traditional stove (e.g., three-stone fire) is replaced with a cleaner-burning improved stove that results in a true exposure reduction of 50%, given the largely overlapping exposure distributions due to the estimated instrument measurement uncertainty (illustrated by the horizontal error bars), the estimated change in ALRI episodes would range from an increase of 6 to a decrease of 26 per 100 children per year (based on estimated incidences of 48–64 per 100 children per year and 38–54 per 100 children per year for traditional and improved stove use, respectively).

Exposure uncertainty that weakens the ability to model exposure–response relationships is not just instrument measurement precision and accuracy, but it is also measured and unmeasured components of the true variability in exposure [e.g., spatial and temporal variability, patterns of stove usage over time, and identification and measurement of the most health-relevant pollutant(s)]. In addition to exposure uncertainty, uncertainty in assessing the health outcome of interest may be similar in magnitude and therefore also weakens the ability to model exposure–response relationships. Depending on the source of the error, the magnitude of uncertainty may vary based on the location along the curve (i.e., at higher or lower exposure concentrations). For example, the amount of instrument measurement uncertainty likely increases with increasing exposure concentrations as hypothesized in Figure 3, whereas uncertainty resulting from health effect estimates is typically larger at both extremes of exposure where sample sizes are often smaller (e.g., Smith et al. 2011). In reality, these sources of error are difficult to distinguish from true variability and result in high levels of uncertainty. Furthermore, information regarding which of the sources of uncertainty are driving the overall exposure uncertainty is lacking and thus constitutes an important research need.

## Conclusions

The rapidly increasing investment in cookstove replacement programs around the world holds promise for addressing a long-standing public health crisis, but it also adds urgency to the need to better characterize the range of health effects related to household air pollution and the risks associated with specific exposure levels. Within this context, it cannot be overstated that more sophisticated approaches to exposure assessment are necessary to address and reduce the complex uncertainty and variability associated with household air pollution exposures, and that increased sophistication will require an increased allocation of study funds for exposure assessment. Reducing the public health impacts from exposure to cookstove emissions hinges on a better understanding of the exposure–response relationship to answer the fundamental question “How clean is clean enough?” Challenges exist in characterizing health outcomes in developing countries, but the historical tendency toward inadequate exposure assessment will not be sufficient to guide the design of improved cookstoves and to understand health benefits and emission reductions of such stove interventions in real-world settings. Of course, extensive exposure assessment for all participants of every health study may not be necessary or even feasible depending on resources and the goal of the study. Instead, at a minimum, coordinated approaches of in-depth exposure characterization nested within larger cookstove studies will leverage research resources. Describing and quantifying the various components of overall exposure error, as well as understanding how patterns of exposure drive the onset and severity of different health outcomes, are key issues for future studies. The answers to the research needs specified above are likely not widely generalizable; the information regarding spatial and temporal variability in exposure will vary by factors such as study location, climate, stove type, fuel characteristics, and home characteristics related to ventilation. Without an improved understanding of this exposure–response relationship, there will continue to be unacceptable uncertainty regarding the level of emissions reduction necessary to meet the health targets of major cookstove replacement interventions. As a result, these interventions may do little to improve health and could represent a squandering of limited resources available for public health programs in developing country settings.
